# Epidermal growth factor-mediated Rab25 pathway regulates integrin β1 trafficking in colon cancer

**DOI:** 10.1186/s12935-018-0526-y

**Published:** 2018-03-05

**Authors:** Kyung Sook Hong, Eun-Young Jeon, Soon Sup Chung, Kwang Ho Kim, Ryung-Ah Lee

**Affiliations:** 1 Department of Surgery and Critical Care Medicine, Ewha Womans University College of Medicine, Seoul, South Korea; 2 Ewha Medical Research Institute, Ewha Womans University College of Medicine, Seoul, South Korea; 3 Department of Surgery, Ewha Womans University College of Medicine, Seoul, South Korea

**Keywords:** Colonic neoplasms, Receptor trafficking, Endocytosis, Shedding, Integrins, Epidermal growth factor, Epidermal growth factor receptor, Rab25, Target therapy

## Abstract

**Background:**

Integrins play a critical role in carcinogenesis. Integrin β1 localization is regulated by the guanosine-5′-triphosphate hydrolase Rab25 and integrin β1 levels are elevated in the serum of colon cancer patients; thus, the present study examined the effects of epidermal growth factor (EGF) and Rab25 on integrin β1 localization in colon cancer cells.

**Methods:**

HCT116 human colon cancer cells were treated with increasing concentrations of EGF, and cell proliferation and protein expression were monitored by MTT and western blot analyses, respectively. Cell fractionation was performed to determine integrin β1 localization in the membrane and cytosol. Integrin β1 extracellular shedding was monitored by enzyme-linked immunosorbent assays (ELISAs) with culture supernatants from stimulated cells. HCT116 cells were transfected with Rab25-specific siRNA to determine the significance of Rab25 in integrin β1 trafficking in the presence of EGF.

**Results:**

Total integrin β1 expression increased in response to EGF and subsequently decreased at 24 h post-stimulation. A similar decrease was observed in purified membrane fractions, whereas no changes were observed in cytosolic levels. ELISAs using media from stimulated cell cultures demonstrated increased integrin β1 levels corresponding to the decrease observed in membrane fractions, suggesting that EGF induces integrin receptor shedding. EGF stimulation in Rab25-knockdown cells resulted in integrin β1 accumulation in the membrane, suggesting that Rab25 promotes integrin endocytosis.

**Conclusions:**

Integrin β1 is shed from colon cancer cells in response to EGF stimulation in a Rab25-dependent manner. These results further the present understanding of the role of integrin β1 in colon cancer progression.

## Background

Integrins are a family of heterodimeric transmembrane proteins composed of α and β subunits that function as bidirectional receptors for extracellular matrix proteins to regulate cell adhesion, motility, and proliferation [1]. These activities are dynamically regulated by receptor internalization and recycling back to the plasma membrane, a process known as trafficking [2–5].

Cancer cells often dissociate from primary tumors and migrate to distant organs and tissues, where they can develop into metastases. This process requires complex interactions with surrounding extracellular matrix proteins or adjacent cells mediated by cell surface receptors [6–9], including integrins [10], The largest subgroup of integrin heterodimers contains integrin β [11, 16], which is overexpressed in solid tumors and is associated with diminished survival [11–15]. Notably, integrin β1 is also detected in the serum of colon cancer patients, where its expression correlates with stage, invasive potential, and the presence of micrometastasis [16].

Integrins have also been shown to modulate epidermal growth factor receptor (EGFR) signaling through various cross-talk mechanisms in an epidermal growth factor (EGF)-dependent or -independent manner [10, 17, 18]. Additionally, EGFR ligation and signaling can promote integrin gene expression to regulate subsequent EGF-EGFR signaling, and extensive crosstalk is reported to occur between EGFR and integrin β1 in breast and lung cancers. Moreover, integrin β1 has been identified as a drug target in several solid tumors [7, 19–22]; thus, further analysis of integrin signaling may have implications for cancer therapy [6, 11]. Because both EGF/EGFR pathway activation and integrin β1 have been associated with colon cancer progression, the present study aimed to investigate changes in integrin β1 expression and trafficking in response to EGF stimulation.

## Methods

### Cell culture and EGF treatment

The HCT116 human colon cancer cell line (No. 10247, Korean Cell Line Bank, Seoul, Korea) was cultured in Roswell Park Memorial Institute (RPMI) 1640 media supplemented with 10% heat-inactivated fetal bovine serum and 100 U/mL penicillin in a 37 °C incubator with 5% CO_2_. Media were replaced three times per week and cells were cultured in serum-free media for 24 h before EGF treatment in all experiments.

### Reagents

RPMI 1640 media and fetal bovine serum (S001-01) were purchased from Wellgene (Gyeongsan-si, Gyeongsangbukdo, Korea). Trypsin-Versene (EDTA) and antibiotics (penicillin–streptomycin) were from Lonza (Basel, Switzerland). Recombinant human EGF (PHG0311) was from Invitrogen (Carlsbad, CA, USA). Thiazolyl blue tetrazolium bromide for MTT assays was purchased from Sigma (St. Louis, MO, USA). Pro-prep lysis buffer for protein extraction was obtained from iNtRON Biotechnology (17081, Seongnam-si, Gyeonggi-do, Korea). Western blot reagents, including 30% acrylamide/bis-acrylamide solution, Tris–HCl (pH 6.8), Tris–HCl (pH 8.8), ammonium persulfate, and 10% sodium dodecyl sulfate (SDS), were from Bio-Rad (Hercules, CA, USA). Mouse anti-CD29 and cytosolic anti-integrin β1 antibodies were purchased from BD Biosciences (San Jose, CA, USA) and Abcam, (Cambridge, UK), respectively. Anti-glyceraldehyde 3-phosphate dehydrogenase (GAPDH, D16H11, rabbit), anti β-actin (13E5, rabbit), and anti Rab25 (D4P6P, rabbit) antibodies were from Cell Signaling Technology (Danvers, MA, USA). All antibodies were diluted 1:1000, except for those against GAPDH and β-actin, which were used at 1:2000. Anti-mouse and anti-rabbit IgG horse radish peroxidase (HRP)-conjugated secondary antibodies were manufactured by Santa Cruz Biotechnology (Dallas, TX, USA).

### MTT assay

HCT116 cells were cultured in 96-well plates and stimulated with increasing concentrations of EGF (1, 10, and 100 ng/mL) for 5, 30 min, 1, 12, 24, or 48 h. Thereafter, thiazolyl blue tetrazolium bromide (MTT, 5 mg/mL in PBS) was added to each well and incubated at 37 °C for another 4 h. The culture medium was then removed from each well, and 60 μL of EtOH:DMSO solution was added to solubilize the formazan crystals. The absorbance at 590 nm was measured using an enzyme-linked immunosorbent assays (ELISA) plate reader.

### Protein purification

Cultured cells were washed with cold PBS twice and treated with trypsin–EDTA for 3 min at 37 °C. Complete medium was added to inactivate trypsin–EDTA, and cells were collected in a tube and centrifuged at 1500 rpm for 3 min. Harvested cell pellets were treated with pro-prep lysis buffer, incubated on ice for 30 min, and centrifuged at 13,000 rpm for 30 min, and then the supernatants were transferred to a new tube.

### Western blot analysis

Proteins for western blot analysis were quantified in Bradford assays with a BSA standard. Samples were separated by 10 or 12% SDS-PAGE, and transferred to polyvinylidene difluoride (PVDF) membranes. After blocking with 5% BSA-TBST at room temperature for 1 h, membranes were incubated with the primary antibody at 4 °C, washed with TBST buffer three times for 5 min each, and then incubated again with the secondary antibody (1:5000) for 1 h. After a final washing, immunoreactive bands were detected with SuperSignal West Pico Chemiluminescent Substrate (Thermo Scientific, Waltham, MA, USA). Densitometric quantification was performed in Image J software (National Institutes of Health, Bethesda, MD, USA).

### ELISA assays

Shed integrin β1 was quantified in culture supernatants using an ELISA kit (Cloud-Clone Corp., Houston, TX, USA). For this, experimental supernatants and reference standards were added to ELISA plates coated with anti-integrin β1 antibody, followed by sequential incubations with biotin-conjugated anti-integrin β1 antibody and HRP-conjugated avidin at 37 °C for 1 h. Thereafter, TMB (3,3′,5,5′-tetramethylbenzidine) colorimetric substrate solution was added and the enzyme–substrate reactions were subsequently terminated by addition of sulfuric acid solution. The absorbance at 450 nm was measured with an ELISA reader.

### Cytosol/membrane fractionation

Cytosolic and membrane fractions were isolated with a Membrane Fractionation Kit (Abcam, AB139409) according to the manufacturer’s instructions. Briefly, harvested HCT116 cells were washed once with Buffer A, and Solvent I was used to permeabilize the plasma membrane selectively. The cytosolic fraction was then isolated from the membranes and nuclei by centrifugation. Membrane fractions were subsequently collected following treatment with Solvent II and a second centrifugation step. The isolated fractions were then analyzed by western blot analysis.

### siRNA transfection

Aliquots containing 8 × 10^5^ HCT116 cells were plated in 60-mm culture plates containing media without antibiotics and incubated overnight. Rab25 FlexTube siRNA (Hs_RAB25_5, Qiagen, Hilden, Germany), RNAiMAX lipofectamine reagent (13778-030, Invitrogen), and Opti-MEM (Invitrogen) were mixed, incubated at room temperature for 20 min, and then added to cells. The transfection medium was replaced after 4 h, and the cells were cultured for additional 24 h before EGF stimulation and harvesting.

### Statistical analysis and Institutional review board statement

Parametric data were analyzed with Student’s *t*-tests. Non-parametric data were analyzed using the Mann–Whitney test. All experiments were repeated at least three times. p < 0.05 was considered statistically significant. The study was reviewed and approved by the Ewha Womans University Mokdong Hospital Institutional Review Board (IRB number: EUMC 2017-06-025).

## Results

### MTT assay

**Fig. 1:**
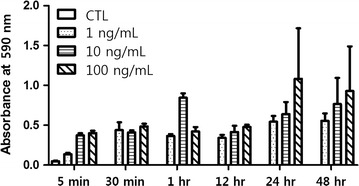
Changes of EGF-treated HCT116 cells. The optimal EGF concentration for future experiments was set at 100 ng/mL, and the optimal EGF exposure time was set at 24 and 48 h mainly (*CTL* control)

EGF concentrations and treatment times were based on previous studies with colon cancer cell lines. Specifically, HCT116 cells were treated with 1, 10, or 100 ng/mL EGF for 5, 30 min, 1, 12, 24, or 48 h, and proliferation was assessed by the MTT assay. These analyses revealed that HCT116 cell proliferation gradually increased in a concentration- and time-dependent manner (Fig. 1).

### Alterations in integrin β1 and Rab25 expression following EGF exposure

**Fig. 2:**
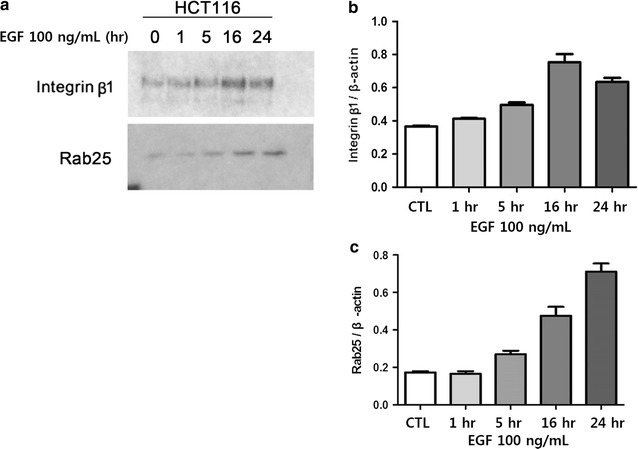
Integrin β1 and Rab25 expression in EGF-treated cells. **a** Integrin β1 and Rab25 expression was examined in HCT116 cells stimulated with 100 ng/mL EGF by western blotting. **b**, **c** Densitometric quantification of the data shown in **a** for **b** integrin β1 and **c** Rab25 (*CTL* control)

**Fig. 3:**
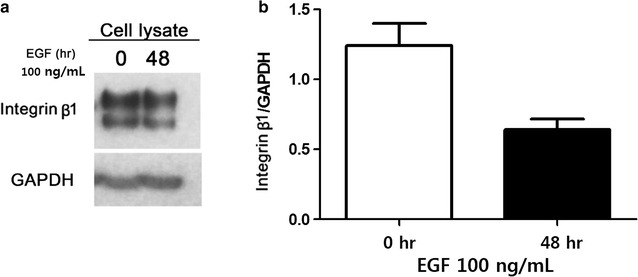
Integrin β1 expression following EGF stimulation for 48 h. **a** Integrin β1 expression following stimulation with 100 ng/mL EGF was monitored by western blotting. **b** Densitometric quantification of the data shown in **a** (p = 0.026)

HCT116 cells were exposed to 100 ng/mL EGF for 24 h, and integrin β1 and Rab25 expression was monitored by western blotting (Fig. 2). Notably, integrin β1 expression increased over time in response to EGF stimulation, peaking at 16 h and decreasing thereafter relative to the β-actin control (p < 0.05; Fig. 2a, b). A similar result was found for Rab25 expression, which also increased in response to EGF treatment (p < 0.05; Fig. 2a, c). Interestingly, prolonged exposure to EGF for 48 h resulted in a significant decrease in integrin β1 expression when compared to basal levels (p = 0.026; Fig. 3).

### Effects of EGF treatment on integrin β1 trafficking and secretion

**Fig. 4:**
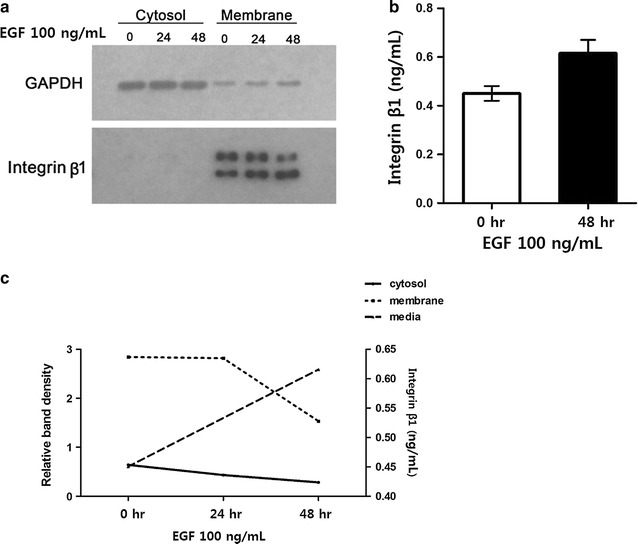
Analysis of integrin β1 localization and shedding. **a** Integrin β1 localization in the membrane and cytosol was examined by subcellular fractionation and western blotting. **b** Integrin β1 shedding was monitored by ELISA after stimulation with EGF for 48 h. **c** EGF-dependent changes in integrin β1 subcellular localization were examined by densitometric quantification of data shown in **a**

To determine whether EGF stimulation altered the localization of integrin β1, HCT116 cells were treated with 100 ng/mL EGF and then subjected to subcellular fractionation and western blot analysis. These results demonstrated that integrin β1 was almost exclusively localized to the membrane fraction, and its expression progressively decreased in response to EGF treatment at 24 and 48 h (p = 0.026; Fig. 4a). Because integrin β1 was not detected in the cytosolic fraction, we performed ELISA analyses with culture media collected after 48 h of exposure to 100 ng/mL EGF. As a result, we found an increase in integrin β1 levels from 0.451 ng/mL in untreated cultures to 0.616 ng/mL after 48 h of EGF treatment (Fig. 4b). Relative changes in integrin β1 localization in the cytosol, membrane, and culture supernatants are shown in Fig. 4c.

### Respective effects of Rab25 expression and EGF stimulation on integrin β1 expression and trafficking

**Fig. 5:**
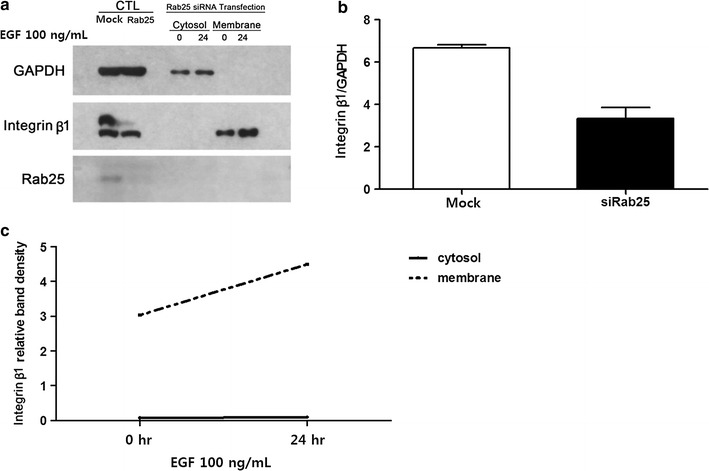
Alterations in integrin β1 localization after Rab25 knockdown. **a** Integrin β1 and Rab25 expression was monitored by western blotting after fractionation. **b** Densitometric quantification of integrin β1 expression in mock and Rab25-knockdown cells. **c** Densitometric quantification of data shown in **a** (*CTL* control)

We next sought to determine whether integrin β1 expression was regulated by Rab25. For this, we transfected HCT116 colon cancer cells with Rab25-specific siRNA and confirmed sufficient knockdown by western blotting (Fig. 5a). Subsequent analysis of integrin β1 levels revealed a significant decrease following Rab25 knockdown (p = 0.003; Fig. 5b). Moreover, membrane/cytosolic fractionation demonstrated that although integrin β1 was still undetectable in the cytoplasm, a marked increase occurred in the membrane fraction after 24 h of EGF treatment (p = 0.001) (Fig. 5c).

**Fig. 6:**
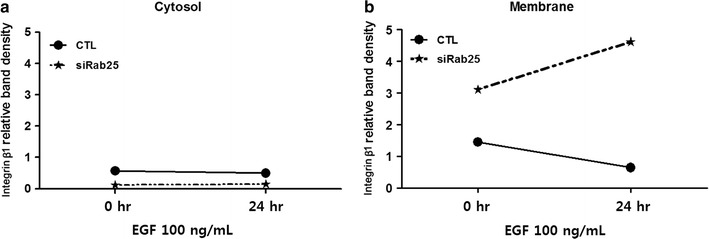
Changes of integrin β1 expression in response to EGF stimulation and Rab25-knockdown. **a**, **b** Relative distribution of integrin β1 in **a** cytosol and **b** membrane following EGF stimulation in control (*CTL* control, solid line) and Rab25-knockdown (siRab25, dotted line) cells

Further densitometric analysis was performed to determine the effects of EGF stimulation and Rab25 expression on integrin β1 localization. Notably, the low levels of integrin β1 in the cytosol were further reduced in response to EGF exposure (p = 0.045), whereas an opposite effect was observed in EGF-treated Rab25 knockdown cells (p = 0.011; Fig. 6a). Additionally, EGF stimulation decreased membrane integrin β1 levels in control cells (p < 0.001); however, this was reversed in Rab25 knockdown cells, where integrin β1 levels in the membrane increased following EGF treatment (p = 0.001) (Fig. 6b).

## Discussion

Colon cancer is highly prevalent and accounts for a large proportion of cancer-related deaths worldwide [23, 24]. Despite recent improvements in survival rates because of advancements in surgical techniques and therapy, metastasis to lymph nodes or distant organs is still a major risk factor for colon cancer death [25]. Malignant progression requires cancer cell proliferation and subsequent invasion into proximal tissues, the latter of which requires complex interactions with proximal extracellular matrix proteins mostly mediated by heterodimeric integrin receptors expressed on the cell surface [6–9]. Mechanistically, integrins regulate cellular adhesion via bidirectional signaling [1] and intercellular crosstalk with other signaling pathways, such as those emanating from growth factor receptors [7].

EGFR is a transmembrane protein that regulates cell proliferation in a tyrosine kinase-dependent manner [26, 27]. Recent studies on putative effectors of EGFR signaling have focused on their functional significance to malignant progression. Thus, integrins are a potential molecular target and/or biomarker for therapeutic resistance because of their known roles in EGFR crosstalk [28]. Specifically, the integrin subunit β1 is reported to regulate signals downstream of the EGFR in breast and lung cancers, and is a putative drug target in several solid tumors [7, 19–22]. Moreover, various modes of crosstalk exist between integrins and EGFR-mediated signaling. For instance, EGFR and integrins can cooperatively induce a single signaling pathway or potentiate EGFR–EGF complex formation, which can result in enhanced integrin expression. Additionally, some integrins also stimulate EGFR pathway activation in the absence of EGF [17].

In the present study, changes of integrin β1 expression and localization were examined in HCT116 colon cancer cells after EGF exposure. These analyses revealed that EGF stimulates integrin β1 expression in a concentration- and time-dependent manner (Fig. 1). Notably, integrin β1 levels gradually increased following stimulation with 100 ng/mL EGF, peaking at 16 h post-treatment (p < 0.05) (Fig. 2).

Integrins connect the extracellular matrix with the actin cytoskeleton. To regulate this activity, integrin heterodimers on the cell surface are continuously endocytosed and recycled back to the plasma membranes to regulate their activity [2, 3, 17, 29]. During metastasis, cancer cells are shed from the primary tumor through variable cell–cell or cell–matrix interactions, or reversible cytoskeletal changes, and subsequently adhere to the extracellular matrix at distant sites where they can invade and develop into metastases. As expected, integrins are significantly involved in these processes, and shedding of integrins reduces the adhesiveness of cancer cells, resulting in higher motility [30]. Consistent with this, the present study determined that EGF stimulation altered the expression and localization of integrin β1. Specifically, total integrin β1 protein levels were markedly lower in HCT116 colon cancer cells treated with EGF for 24 h (Figs. 2, 3). This finding was entirely specific to membrane integrin β1 expression, as no significant changes were observed in the cytosolic fraction (Fig. 4). Further analysis of integrin β1 levels in the culture medium by ELISA revealed a corresponding increase in soluble integrin β1 (Fig. 4), suggesting that the decrease in membrane protein was the result of integrin shedding [30]. Collectively, these data suggest that EGF stimulation promotes integrin expression and shedding into the extracellular space, thereby regulating cell–cell interactions and cell motility, respectively.

Rab family proteins are low-molecular-weight GTP hydrolases with important functions in the intracellular trafficking of integrins [2, 4, 5, 31–33]. Rab25 expression is associated with colon cancer progression [34–37], and was thus investigated in the present study. Notably, siRNA-mediated Rab25 knockdown resulted in an overall reduction of integrin β1 levels (Fig. 5). Moreover, significantly lower levels of cytosolic integrin β1 were found in knockdown cells, but cytosolic integrin β1 levels were unchanged in response to EGF exposure, whereas a marked accumulation was observed in the membrane (Fig. 5). Densitometric analysis of integrin β1 localization in response to Rab25 knockdown and/or EGF stimulation confirmed a reduced level of cytosolic integrin β1 in Rab25 knockdown cells that was not changed by EGF exposure overall (Fig. 6a), whereas membrane levels increased after Rab25 inhibition and accumulated with subsequent EGF treatment (Fig. 6b), suggesting that Rab25 altered the cellular distribution of integrin β1. Considering that Rab proteins are involved in intracellular protein trafficking [2, 4, 5, 31–33], these data support a mechanism in which EGF and Rab25 promote integrin β1 expression and endocytosis, respectively.

Many recent cancer studies have focused on the development of molecular targeted therapies. Although EGF–EGFR signaling is only one of several pathways involved in cancer cell proliferation and motility, the respective effects of EGF and Rab25 on integrin localization revealed in the present study may partially explain the diverse effects of EGFR-targeting drugs. Nevertheless, further studies on the respective effects of EGF, integrin β1, and Rab25 in the progression and metastasis of colon cancer would enable the therapeutic application of these drugs.

In summary, EGF stimulation increases integrin β1 expression in colon cancer cells and may regulate its cellular localization in a Rab25-dependent manner; however, continued investigation into these processes would further delineate the functional significance of these factors in colon cancer progression.

## Conclusions

Integrin β1 is recycled by trafficking and shed from colon cancer cells in response to EGF stimulation in a Rab25-dependent manner. These results further the understanding of the role of integrin β1 in colon cancer progression.

## Abbreviations

EGF: epidermal growth factor
EGFR: epidermal growth factor receptor
ELISA: enzyme-linked immunosorbent assay
HRP: horse radish protein
PVDF: polyvinylidene difluoride
SDS: sodium dodecyl sulfate
RPMI: Roswell Park Memorial Institute

## References

[1] Hynes RO (2002). Integrins: Bidirectional, Allosteric Signaling Machines. Cell.

[2] Caswell PT, Norman JC (2006). Integrin trafficking and the control of cell migration. Traffic..

[3] Jones MC, Caswell PT, Norman JC (2006). Endocytic recycling pathways: emerging regulators of cell migration. Curr Opin Cell Biol.

[4] Nielsen E, Cheung AY, Ueda T (2008). The regulatory RAB and ARF GTPases for vesicular trafficking. Plant Physiol.

[5] Wang C, Yoo Y, Fan H, Kim E, Guan KL, Guan JL (2010). Regulation of Integrin β1 recycling to lipid rafts by Rab1a to promote cell migration. J Biol Chem.

[6] Desgrosellier JS, Cheresh DA (2010). Integrins in cancer: biological implications and therapeutic opportunities. Nat Rev Cancer.

[7] Bernardes N, Abreu S, Carvalho FA, Fernandes F, Santos NC, Fialho AM (2016). Modulation of membrane properties of lung cancer cells by azurin enhances the sensitivity to EGFR-targeted therapy and decreased β1 integrin-mediated adhesion. Cell Cycle.

[8] Streuli CH, Akhtar N (2009). Signal co-operation between integrins and other receptor systems. Biochem J.

[9] Rathinam R, Alahari SK (2010). Important role of integrins in the cancer biology. Cancer Metastasis Rev.

[10] Blandin AF, Renner G, Lehmann M, Lelong-Rebel I, Martin S, Dontenwill M (2015). β1 integrins as therapeutic targets to disrupt hallmarks of cancer. Front Pharmacol.

[11] Schaffner F, Ray AM, Dontenwill M (2013). Integrin α5β1, the fibronectin receptor, as a pertinent therapeutic target in solid tumors. Cancers (Basel).

[12] Paulus W, Baur I, Schuppan D, Roggendorf W (1993). Characterization of integrin receptors in normal and neoplastic human brain. Am J Pathol.

[13] Barkan D, Chambers AF (2011). β1-integrin: a potential therapeutic target in the battle against cancer recurrence. Clin Cancer Res.

[14] Fabricius EM, Wildner GP, Kruse-Boitschenko U, Hoffmeister B, Goodman SL, Raguse JD (2011). Immunohistochemical analysis of integrins αvβ3, αvβ5 and α5β1, and their ligands, fibrinogen, fibronectin, osteopontin and vitronectin, in frozen sections of human oral head and neck squamous cell carcinomas. Exp Ther Med.

[15] Lahlou HR, Muller WJ (2011). β1-integrins signaling and mammary tumor progression in transgenic mouse models: implications for human breast cancer. Breast Cancer Res.

[16] Oh BY, Kim KH, Chung SS, Hong KS, Lee RA (2014). Role of β1-integrin in colorectal cancer: case–control study. Ann Coloproctol.

[17] Ivaska J, Heino J (2011). Cooperation between integrins and growth factor receptors in signaling and endocytosis. Annu Rev Cell Dev Biol.

[18] Moro L, Dolce L, Cabodi S, Bergatto E, Boeri Erba E, Smeriglio M, Turco E, Retta SF, Giuffrida MG, Venturino M, Godovac-Zimmermann J, Conti A, Schaefer E, Beguinot L, Tacchetti C, Gaggini P, Silengo L, Tarone G, Defilippi P (2002). Integrin-induced epidermal growth factor (EGF) receptor activation requires c-Src and p130Cas and leads to phosphorylation of specific EGF receptor tyrosines. J Biol Chem.

[19] Morello V, Cabodi S, Sigismund S, Camacho-Leal MP, Repetto D, Volante M, Papotti M, Turco E, Defilippi P (2011). β1 integrin controls EGFR signaling and tumorigenic properties of lung cancer cells. Oncogene.

[20] Ju L, Zhou C (2013). Integrin β1 enhances the epithelial-mesenchymal transition in association with gefitinib resistance of non-small cell lung cancer. Cancer Biomark.

[21] Zhang X, Fournier MV, Ware JL, Bissell MJ, Yacoub A, Zehner ZE (2009). Inhibition of vimentin or β1 integrin reverts morphology of prostate tumor cells grown in laminin-rich extracellular matrix gels and reduces tumor growth in vivo. Mol Cancer Ther.

[22] Carpenter PM, Dao AV, Arain ZS, Chang MK, Nguyen HP, Arain S, Wang-Rodriguez J, Kwon SY, Wilczynski SP (2009). Motility induction in breast carcinoma by mammary epithelial laminin 332 (laminin 5). Mol Cancer Res.

[23] Jemal A, Bray F, Center MM, Ferlay J, Ward E, Forman D (2011). Global cancer statistics. CA Cancer J Clin.

[24] Siegel R, Desantis C, Jemal A (2014). Colorectal cancer statistics, 2014. CA Cancer J Clin.

[25] Steinert G, Scholch S, Koch M, Weitz J (2012). Biology and significance of circulating and disseminated tumour cells in colorectal cancer. Langenbecks Arch Surg.

[26] Manning BD, Cantley LC (2007). AKT/PKB signaling: navigating downstream. Cell.

[27] Normanno N, Bianco C, Strizzi L, Mancino M, Maiello MR, De Luca A, Caponigro F, Salomon DS (2005). The ErbB receptors and their ligands in cancer: an overview. Curr Drug Targets.

[28] Scartozzi M, Giampieri R, Loretelli C, Mandolesi A, del Prete M, Biagetti S, Alfonsi S, Faloppi L, Bianconi M, Bittoni A, Bearzi I, Cascinu S (2013). Role of β4 integrin in HER-3-negative, K-RAS wild-type metastatic colorectal tumors receiving cetuximab. Future Oncol.

[29] Pellinen T, Ivaska J (2006). Integrin traffic. J Cell Sci.

[30] Kryczka J, Stasiak M, Dziki L, Mik M, Dziki A, Cierniewski C (2012). Matrix metalloproteinase-2 cleavage of the β1 integrin ectodomain facilitates colon cancer cell motility. J Biol Chem.

[31] Mellman I, Nelson WJ (2008). Coordinated protein sorting, targeting and distribution in polarized cells. Nat Rev Mol Cell Biol.

[32] Schwartz SL, Cao C, Pylypenko O, Rak A, Wandinger-Ness A (2007). Rab GTPases at a glance. J Cell Sci.

[33] Caswell PT, Spence HJ, Parsons M, White DP, Clark K, Cheng KW, Mills GB, Humphries MJ, Messent AJ, Anderson KI (2007). Rab25 associates with α5β1 integrin to promote invasive migration in 3D microenvironments. Dev Cell.

[34] Nam KT, Lee HJ, Smith JJ, Lapierre LA, Kamath VP, Chen X, Aronow BJ, Yeatman TJ, Bhartur SG, Calhoun BC, Condie B, Manley NR, Beauchamp RD, Coffey RJ, Goldenring JR (2010). Loss of Rab25 promotes the development of intestinal neoplasia in mice and is associated with human colorectal adenocarcinomas. J Clin Invest.

[35] Goldenring JR, Nam KT (2011). Rab25 as a tumour suppressor in colon carcinogenesis. Br J Cancer.

[36] Krishnan M, Lapierre LA, Higginbotham JN, Goldenring JR (2011). RAB25 regulates polarity in intestinal epithelial cells. Mol Biol Cell.

[37] Krishnan M, Lapierre LA, Knowles BC, Goldenring JR (2013). Rab25 regulates integrin expression in polarized colonic epithelial cells. Mol Biol Cell.

